# A globally diverse reference alignment and panel for imputation of mitochondrial DNA variants

**DOI:** 10.1186/s12859-021-04337-8

**Published:** 2021-09-01

**Authors:** Tim W. McInerney, Brian Fulton-Howard, Christopher Patterson, Devashi Paliwal, Lars S. Jermiin, Hardip R. Patel, Judy Pa, Russell H. Swerdlow, Alison Goate, Simon Easteal, Shea J. Andrews, for the Alzheimer’s Disease Neuroimaging Initiative

**Affiliations:** 1 John Curtin School of Medical Research, Australian National University, Australian Capital Territory, Canberra, Australia; 2 Genetics and Genomic Sciences, Ronald M. Loeb Center for Alzheimer’s Disease, Icahn School of Medicine at Mount Sinai, 1 Gustave L. Levy Place, New York, NY 10029 USA; 3 Keck School of Medicine, Mark and Mary Stevens Neuroimaging and Informatics Institute, University of Southern California, Los Angeles, CA USA; 4 Department of Neurology, Alzheimer’s Disease Research Center, Keck School of Medicine, University of Southern California, Los Angeles, CA USA; 5 CSIRO Land and Water, Commonwealth Scientific Industrial and Research Organization, Acton, ACT 2601 Australia; 6 Research School of Biology, Australian National University, Canberra, ACT 2601 Australia; 7 School of Biology and Environmental Science, University College Dublin, Belfield, Dublin 4, Ireland; 8 Earth Institute, University College Dublin, Belfield, Dublin 4, Ireland; 9 Department of Neurology, Alzheimer’s Disease Center, University of Kansas, Fairway, KS USA

**Keywords:** Mitochondrial DNA, Imputation, Reference panel

## Abstract

**Background:**

Variation in mitochondrial DNA (mtDNA) identified by genotyping microarrays or by sequencing only the hypervariable regions of the genome may be insufficient to reliably assign mitochondrial genomes to phylogenetic lineages or haplogroups. This lack of resolution can limit functional and clinical interpretation of a substantial body of existing mtDNA data. To address this limitation, we developed and evaluated a large, curated reference alignment of complete mtDNA sequences as part of a pipeline for imputing missing mtDNA single nucleotide variants (mtSNVs). We call our reference alignment and pipeline MitoImpute.

**Results:**

We aligned the sequences of 36,960 complete human mitochondrial genomes downloaded from GenBank, filtered and controlled for quality. These sequences were reformatted for use in imputation software, IMPUTE2. We assessed the imputation accuracy of MitoImpute by measuring haplogroup and genotype concordance in data from the 1000 Genomes Project and the Alzheimer’s Disease Neuroimaging Initiative (ADNI). The mean improvement of haplogroup assignment in the 1000 Genomes samples was 42.7% (Matthew’s correlation coefficient = 0.64). In the ADNI cohort, we imputed missing single nucleotide variants.

**Conclusion:**

These results show that our reference alignment and panel can be used to impute missing mtSNVs in existing data obtained from using microarrays, thereby broadening the scope of functional and clinical investigation of mtDNA. This improvement may be particularly useful in studies where participants have been recruited over time and mtDNA data obtained using different methods, enabling better integration of early data collected using less accurate methods with more recent sequence data.

**Supplementary Information:**

The online version contains supplementary material available at 10.1186/s12859-021-04337-8.

## Background

Variation in mitochondrial DNA (mtDNA) is of interest because it is informative about human evolution [1] and because it is associated with numerous human diseases [2]. Because human mitochondrial genomes do not recombine, the relationships among them can be described by a single phylogenetic tree. They can thus be grouped by the phylogenetic lineages to which they belong, into so-called haplogroups. In this system of evolutionary classification, genomes that belong to deeply divergent lineages form major haplogroups, with minor haplogroups corresponding to more recently diverged lineages [3, 4].

In some studies, mtDNA is not fully characterised by whole genome sequencing, but rather by single nucleotide variants (mtSNVs) identified at predetermined sets of mitochondrial genome sites using microarrays [5]. Partial mtSNV data obtained using such arrays may be insufficient for reliable haplotype assignment of mitochondrial genomes. Reliable classification of mtSNV data is important because haplogroup classification is often used in population genetic studies and clinical investigations of associations between mitochondrial genomes and disease [6].

In addition, not all microarrays are designed to assay variation at the same sites in the human mitochondrial genome. Inconsistencies in the design of microarrays used in different studies can result in mtSNV datasets that are partially incompatible, making it difficult to combine them for joint analysis. Legacy datasets lacking whole-genome sequences can be resequenced to provide complete information for adequate comparison. However, given the 40 + years of research of mtDNA, it is probable that the raw biomaterial for many studies are no longer available. Additionally, studies as recent as 2021 have used imputation as a tool to fill in missing mtSNVs for genotype–phenotype association studies [7], highlighting the continued importance imputation plays in biomedical research.

The dual problems of inaccurate haplotype assignment and incompatibility of data from studies that use different microarrays can be resolved by imputing mtSNVs at missing sites from a representative reference panel of human mitochondrial genome sequences. For incomplete mitochondrial genome sequence data, the base states (A, C, G, T) of missing nucleotide sites can be imputed by estimating their probabilities from the co-occurrence, as haplotypes, of bases at sites for which data are available. Accurate estimation of these probabilities has two fundamental requirements: (1) An accurate multiple sequence alignment (MSA) of genome sequences; and (2) A reference panel of genome sequences that is representative of the population being investigated.

The sequences of mitochondrial genomes vary substantially among human populations. To be representative, genome reference data must be obtained from the population that is the target of investigation. Data that is unrepresentative because it was obtained from an inappropriate population, can cause imputation to be biased and inaccurate [8–13]. Additional bias and inaccuracy may arise during construction of MSAs, which entails inserting alignment gaps (‘–’) between some of the nucleotides in some of the sequences being aligned—doing so accurately is a nontrivial challenge [14–16].

Imputation has been used to identify missing nuclear genome variants in incomplete sequence data using the 1000 Genomes Project dataset [17, 18]. However, this dataset, which contains 2504 nuclear and mitochondrial genome sequences representing 26 populations is only partially representative of human genome variation, with some populations (e.g., Pacific Islanders, Indigenous Australians, and Central Asians) still not represented. Thus, to be able to accurately impute missing variants from other populations reference panels containing high-quality complete data from as many globally diverse populations is required.

In addition, considerable work is required to convert the 1000 Genomes Project mtSNV data from the format in which it has been made publicly available to a format that can be used for imputation. There is no published MSA of mitochondrial genomes from the 1000 Genome Project data or other more limited datasets (e.g., [6, 19]) that have been used for mitochondrial genome imputation. In addition to introducing errors, the need to recreate reference panels and MSAs for new studies results in a lack of the standardisation needed for comparison of results from different studies.

Imputation of mtDNA data would be greatly simplified and the substantial existing datasets of incomplete mitochondrial genome sequences would be made more accessible by overcoming the need for: preliminary data reformatting, identification and curation of suitable reference data panels, and standardisation of high-quality multi-sequence alignments.

Here we address these challenges by creating a large (*n* = 36,960) globally diverse MSA using automated alignment software and manual curation by experienced researchers. This resource is publicly available on GitHub as a standard reference MSA of complete sequences in FASTA format (henceforth, the ‘Reference MSA’). We also include the standard Reference MSA only including filtered variable sites in a format readily readable by IMPUTE2 [20] (henceforth, the ‘Reference Panel’). In addition, we describe a SnakeMake pipeline, which we developed for easy imputation of mtSNVs through the IMPUTE2 framework [20]. We call our combined Reference MSA/Panel and pipeline MitoImpute. Finally, we report our evaluation of MitoImpute using in silico microarrays (‘microarray datasets’) derived from The 1000 Genomes Project Consortium [17] whole-genome sequence (WGS) data, and empirical data from the Alzheimer’s Disease Neuroimaging Initiative (ADNI) [21].

## Methods

### Reference alignment and reference panel

Whole human mtDNA sequences were downloaded from GenBank on 18 July 2018 by adapting the MitoMap [22] search term (Additional file 1: Supplementary Methods). This search returned 44,299 complete human mtDNA sequences and excluded archaic and ancient sequences. A curated alignment created in 2011 using 7747 complete mtDNA sequences, which was aligned using a combination of MAFFT and manual decisions on gap character-state placement by experienced researchers (unpublished, Additional file 1: Suppl. Methods; Easteal and Jermiin, pers. comm.). To retain the placement of gap character-states, sequences were aligned to this pre-existing alignment (unpublished, Additional file 1: Suppl. Methods) in batches of 2500 using MAFFT [23] using default settings in Geneious v10.2.6 [24]. The standardised site-numbering convention was maintained by including the revised Cambridge Reference Sequence (rCRS) [25] in both pre-existing and new Reference MSAs, and by removing sites that introduced gap character-states in the rCRS. We considered retaining these sites that introduced gap character-states as they likely represent real insertion events. However, we prioritised a reference alignment that maintained the rCRS site numbering convention. Gaps in sequences that were not the rCRS were retained.

To improve the quality of the Reference MSA, sequences with ≥ 5 ambiguous characters or ≥ 8 gaps were removed. This threshold was set to enable the inclusion of haplogroup B sequences, which averaged 7 gaps relative to other sequences. This quality filter reduced the Reference MSA to 36,960 sequences (Additional file 2: Table 1). To avoid adding bias to population frequency estimates, GenBank accessions with identical sequences were retained on the basis that they represent relatively common mitochondrial genomes. AliStat v1.11 [26] was used to quantify the completeness of the new Reference MSA.

**Fig. 1:**
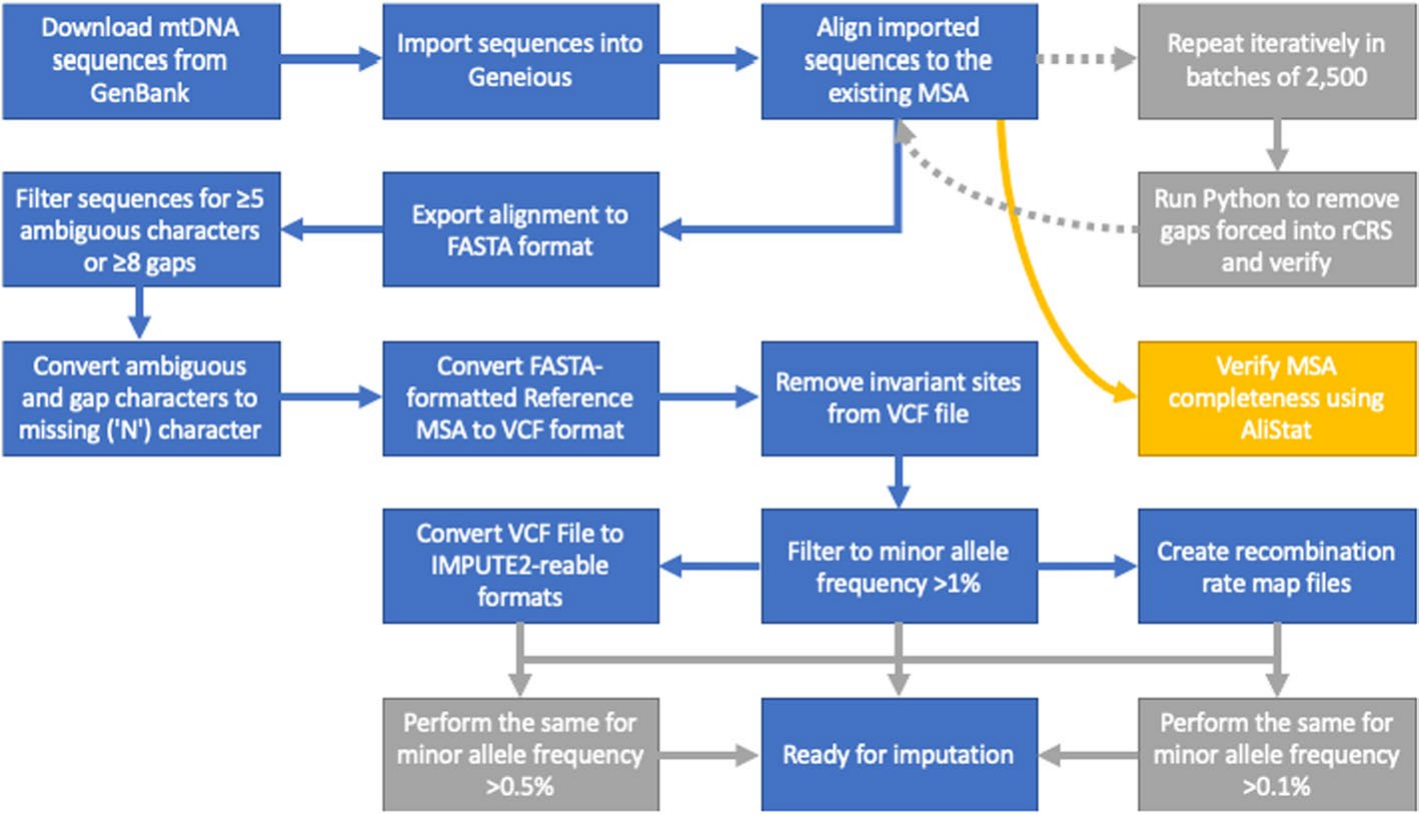
Flowchart of the creation and curation of the reference multiple sequence alignment and reference panel

The Reference Panel was created by converting the Reference MSA to formats compatible with IMPUTE2 [20]. First, the entire Reference MSA was converted into a VCF file with all gap and other ambiguous character-states coded as ‘N’s. Second, all invariant sites were removed from the VCF file using BCFtools v1.4 [27]. Third, the VCF file was converted to the IMPUTE2-readable .gen, .hap, .legend, and .sample files using BCFtools v1.4 [27], and into .ped files using PLINK v1.9 [28]. Finally, recombination map files were created by extracting the site list from the VCF file using BCFtools v1.4 [27] and assigning each site a recombination rate of zero. Furthermore, we created different versions of the Reference Panel by further filtering the VCF to minor allele frequencies of > 1%, > 0.5%, and > 0.1%. This resulted in the Reference Panel having four versions at different minor allele frequency filtering thresholds. We used these thresholds to test how well the imputation procedure performs at for different allele frequency cut-offs; thus, we refer to all four collectively simply as the Reference Panel. A flowchart of the creation and curation of the Reference MSA and Reference Panel is presented in Fig. 1.

### Validation panel

In silico microarrays (‘microarray’ datasets) were created by selecting only mtSNVs present in commercially available microarrays from the 1000 Genomes Project Phase 3 WGS data (*n* = 2535). Microarray information was obtained from strand orientation files available from the Wellcome Centre for Human Genetics at the University of Oxford [29], with 103 strand files containing mtSNVs (Additional file 2: Table 2). Haplogroup assignment for the WGS data and the microarray datasets was performed using HaploGrep2 [30] and Hi-MC [31].

### Imputation

We used the IMPUTE2 X-chromosome imputation protocol [6, 20] to impute missing variants to the microarray datasets from the Reference Panel. No recombination was assumed (i.e., a uniform recombination rate of *r* = 0 across all sites). The Markov chain Monte Carlo step in IMPUTE2, which is used to account for phase uncertainty in recombining diploid data [20], was not used because human mitochondrial genomes are haploid and are not known to recombine. Only high-quality imputed sites were retained by removing sites with an IMPUTE2 information score of ≤ 0.3.

The *k*_*hap*_ parameter in IMPUTE2 specifies the *k* number of haplotypes from the Reference Panel IMPUTE2 will use in the imputation pipeline. The effect of varying the *k*_*hap*_ parameter was assessed by running the imputation pipeline with *k*_*hap*_ set to 100, 250, 500, 1000, 2500, 5000, 10,000, 20,000, and 30,000.

We tested how accurately our imputation pipeline imputes rare variants when the Reference Panel is filtered at different minor allele frequency (MAF) thresholds. We tested thresholds of MAF > 1%, MAF > 0.5% and MAF > 0.1%, resulting in 409, 682 and 1874 mtSNVs, respectively (Additional file 2: Table 3). With this filtering scheme, two of the 103 strand files did not include any mtSNVs at MAF > 1% or MAF > 0.5% (Additional file 2: Table 2). Imputation accuracy was assessed using Matthews Correlation Coefficient (MCC) [32, 33] for genotype concordance. We also assessed imputation accuracy using haplogroup concordance. Both HaploGrep2 [30] and Hi-MC [31] were used for haplogroup assignment, with the complete 1000 Genomes Project WGS data used as the truth set. HaploGrep2 has the advantage of covering the full scope of the PhyloTree haplogroup nomenclature [30, 34], including small sub-haplogroups. Hi-MC was developed for epidemiological research that uses high-throughput data by reducing PhyloTree nomenclature to 46 common haplogroups using a limited array of mtSNVs from which to assign haplogroups. We treated the first major sub-haplogroup of all L linages (i.e., L0), as well as HV and JT as macrohaplogroups [3, 4].

Linear mixed-model ANOVA was used to assess the meaningfulness of difference in MCC (mean of mtSNVs per microarray dataset) and haplogroup assignment for different parameters tested for *k*_*hap*_ and MAF.

**Fig. 2:**
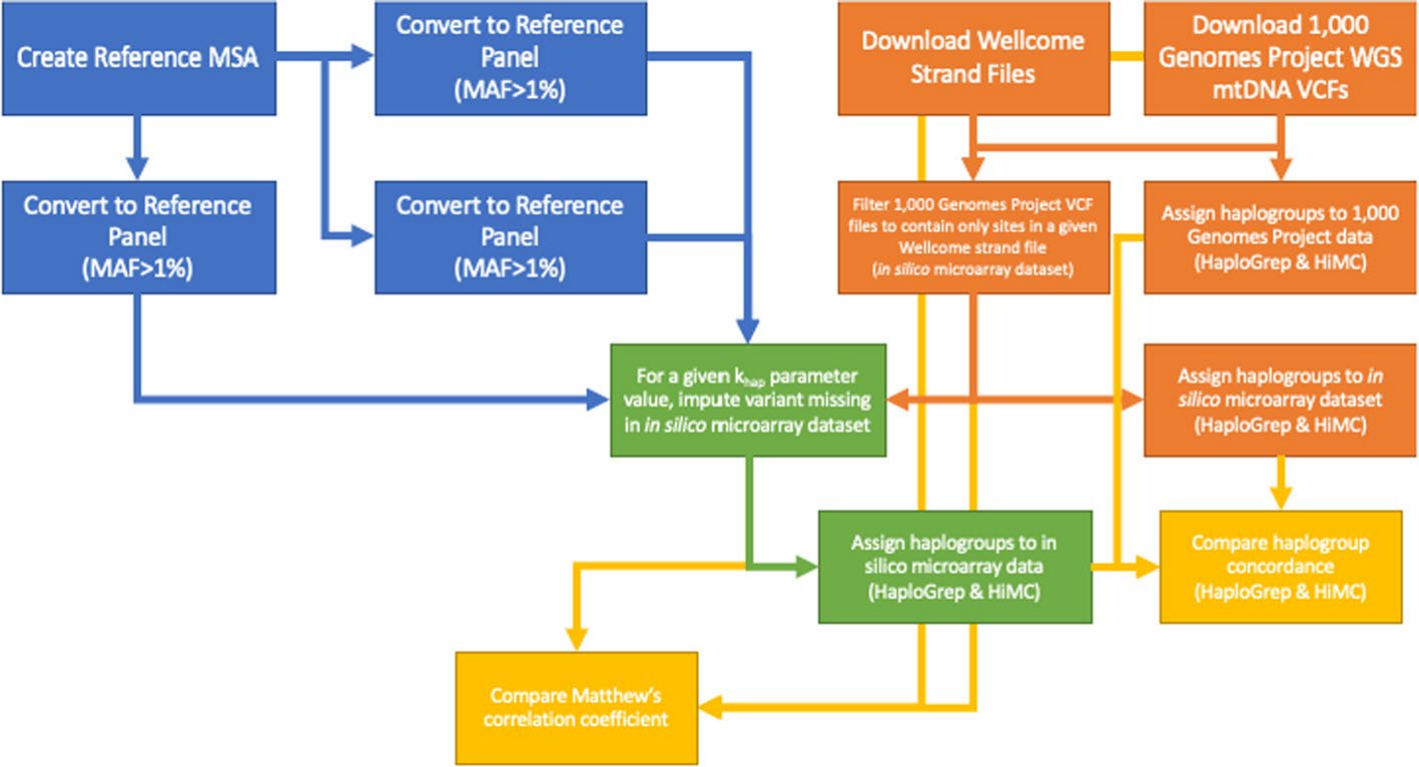
Flowcharts of the MitoImpute imputation and analytical pipelines

Pipelines for implementing our imputation pipeline and reproducing our results were initially created in BASH shell scripts then lifted over into SnakeMake [35] for the MitoImpute pipeline. A flowchart of the imputation and analytical pipeline is presented in Fig. 2.

## Results

### Reference alignment and reference panel

To comply with minimum reporting standards for MSAs, completeness metrics of the Reference MSA were computed (Table 1). As described in Wong, Kalyaanamoorthy [26], *C*_*a*_ is the completeness of the MSA, *C*_*r*_ is the completeness of the *r*th sequence, *C*_*c*_ is the completeness of the *c*th site, and *C*_*ij*_ is the completeness of the *i*th and *j*th sequences. Overall, the Reference MSA is highly complete (*C*_*a*_ > 0.99). Individual sequences are also mostly complete (*C*_*r*_), with the least complete sequence containing completely-specified nucleotides at 91% of its sites and the most complete sequence containing completely-specified nucleotides at all of its sites. The least complete site in the MSA contained completely-specified nucleotides in 44.3% of sequences, and the most complete sites had completely-specified nucleotides in all of the sequences. The proportion of homologous sites with completely-specified nucleotides at sites in both sequences (*C*_*ij*_) ranged from 83 to 100%, suggesting that the majority of sequence pairs contain enough information to quantify evolutionary distances. Sites and sequences missing a substantial number of nucleotide states were removed in the filtration processes as described in the “Methods” section.

**Table 1:** AliStat completeness metrics for the Reference MSA

Feature	Value(s)
Sequences	44,299
Sites	16,569
Completeness score (*C*_*a*_)	0.9997
*C*-score for individual sequences (*C*_*r*_) [min–max]	0.9119–1.0000
*C*-score for individual sites (*C*_*c*_) [min–max]	0.4429–1.0000
*C*-score for pairs of sequences (*C*_*ij*_, *i* ≠ *j*) [min–max]	0.8314–1.0000

C_a_: completeness of the alignment; C_r_: completeness of the rth sequence; C_c_; completeness of the cth site; and C_ij_: completeness of the ith and jth sequences

GenBank metadata on geographic provenance was available for 7128 (19.3% filtered and 16.1% unfiltered) sequences in the Reference Panel, from 49 countries and 54 sub-country regions (Additional file 2: Supplementary Table 4). These regions included smaller ethnic groups such as Yami Taiwanese, Moroccan Berbers, Pacific Islanders, Indigenous Australians, and people from Central Asia and Siberia. For sequences with provenance information, there is, however, a distinct bias towards Europe (3855; 54.1%; 10.4% filtered; 8.7% unfiltered) and East Asia (2065; 29.0%; 5.6% filtered; 4.7% unfiltered).

**Fig. 3:**
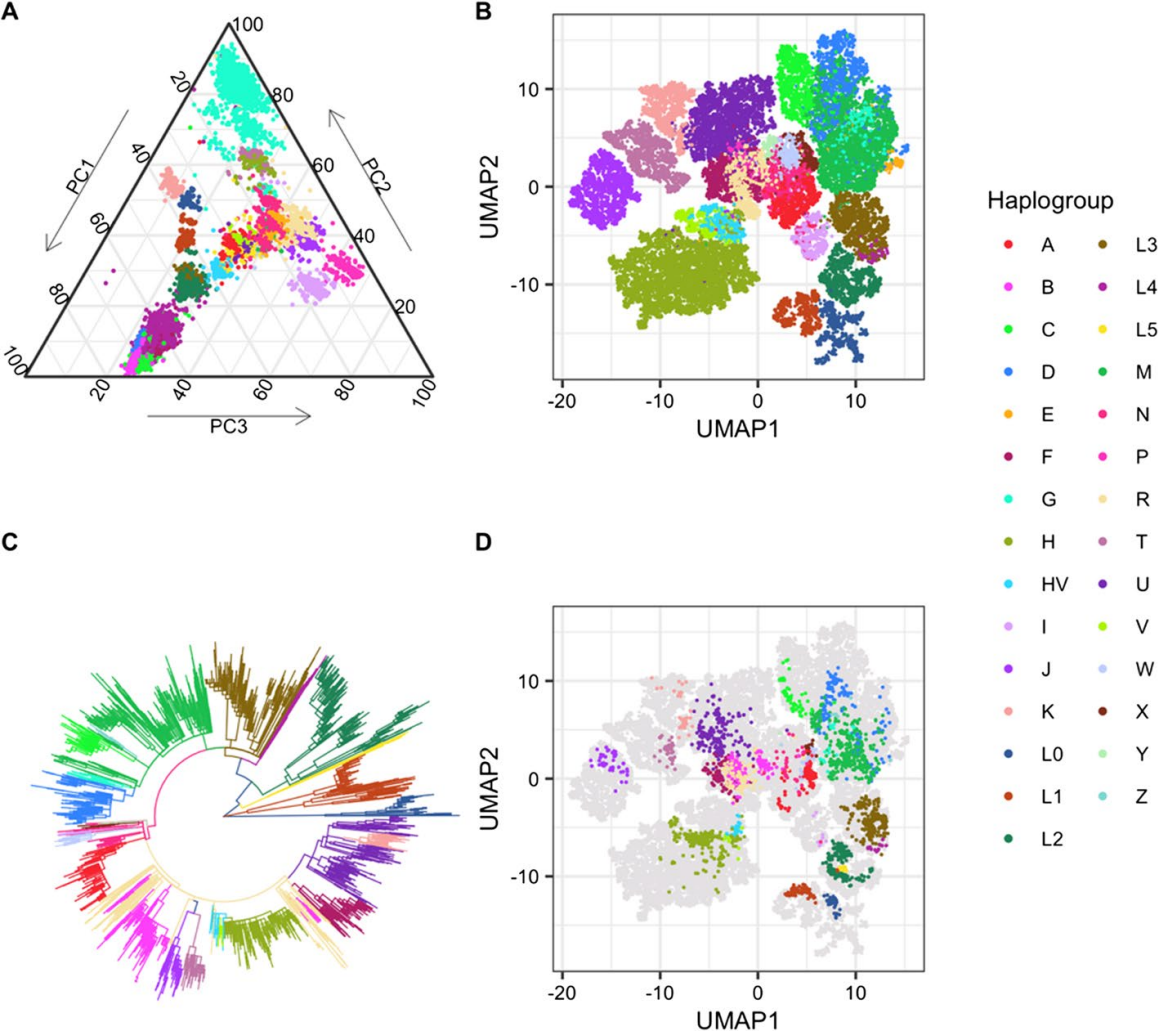
Diversity of mitochondrial Reference Multiple Sequence Alignment. **a** Principal Component Analysis (PCA) of mitochondrial sequencies included in the Reference Panel coloured by haplogroup. **b** Uniform Manifold Approximation and Projection (UMAP) projection of mitochondrial sequences. **c** Phylogenetic tree of 1000 genomes mitochondrial sequences highlighting phylogenetic relationship between mitochondrial haplogroups. **d** Projection of 1000 Genomes mitochondrial sequences onto the mitochondrial reference alignment

All major haplogroups are represented in the Reference Panel (Additional file 1: Table 1), including rare haplogroups such as haplogroup S, which is endemic to Indigenous Australians, haplogroup L5, which is found in Mbuti Pygmies, haplogroup L6, which is found in low frequencies in Yemen and Ethiopia, and haplogroups O and Q, which are found exclusively in the Pacific Islands (Fig. 3). Haplogroup B was the haplogroup most frequently removed by the quality control filter (3395 or 46% of all 7339 removed sequences), leaving only 273 haplogroup B sequences. Haplogroup H was also heavily filtered following quality control (1376; 19%), but remained well represented in the final Reference Panel (n = 7644). Only a small fraction of other haplogroups were removed during quality control.

### In silico microarrays

#### Parameter tuning

We measured imputation accuracy of genotypes using the Matthews Correlation Coefficient (MCC) [32, 33]. To summarise MCC values, we calculated the mean MCC across all imputed sites, then compared the estimated marginal means using a linear mixed-model ANOVA. Our results show that the Reference Panel filtered to MAF > 0.1% was the best performing (${\mu }_{MCC}=0.60$), followed by MAF > 0.5 (${\mu }_{MCC}=0.58$), then by MAF > 1% (${\mu }_{MCC}=0.57$). These contrasts are all statistically significant (ANOVA, $p=0.002$) (Additional file 2: Table 5a-c). For the *k*_*hap*_ parameter, there was no significant pairwise differences between ${k}_{hap}=100$ and the other ${k}_{hap }\mathrm{values up to} 1000$. Above a ${k}_{hap}=1000$, contrasts were often statistically significant (Additional file 2: Table 5d-f), with larger *k*_*hap*_ parameter values performing comparatively poorly, indicating a reduced ability to correctly assign haplogroups for some microarray datasets.

Imputation accuracy was also evaluated using the IMPUTE2 Info Score using the same statistical framework described for MCC. In contrast to MCC, the Reference Panel filtered to MAF > 1% was the best performing (${\mu }_{info}=0.73$), followed by MAF > 0.5 (${\mu }_{MCC}=0.69$), and MAF > 0.1% (${\mu }_{MCC}=0.63$). All of these contrasts are statistically significant (ANOVA, $p<0.0001$) (Additional file 2: Table 6a-c). Starting at ${k}_{hap}=1000$, pairwise comparison of larger *k*_*hap*_ values become statistically significant, suggesting a meaningful difference in mean haplogroup concordance becomes apparent when more reference haplotypes are included.

Imputation accuracy was further evaluated by determining whether haplogroup assignments were concordant between imputed sequenced datasets. As HaploGrep2 assigns haplotypes to very specific sub-haplogroups, we measured concordance using the sub-haplogroups in addition to macrohaplogroups. We found that sub-haplogroup concordance decreased slightly for MAF > 1% (− 2.5%) and MAF > 0.5% (− 0.6%), and only slightly increased using MAF > 0.1% (1.4%). Statistical significance is observed between all these comparisons (Additional file 2: Table 7a-c). The differences between *k*_*hap*_ parameters settings were more pronounced, with all *k*_*hap*_ parameter values showing a decrease in concordance (Additional file 2: Table 7d-f), likely because all *k*_*hap*_ experiments used the Reference Panel filtered at MAF > 1%. Larger *k*_*hap*_ parameter values performed more poorly than smaller values.

Macrohaplogroup concordance increased only slightly following imputation. There was no statistically significant difference between any of the MAF thresholds, although there was a slight increase in accuracy with decreasing MAF (0.8% to 2.2%, ANOVA $p=0.09$). Reference haplotype parameter values from ${k}_{hap}=100$ to ${k}_{hap}=\mathrm{1,000}$ exhibit minor increases in performance, with larger *k*_*hap*_ parameter values leading to relatively poorer imputation performance (Additional file 2: Table 8). We note, however, that the mean macrohaplogroup concordance in the microarray dataset was already > 86.7%.

Additionally, we evaluated whether the HaploGrep2 haplogroup quality score improved following imputation. There was no significant difference in haplogroup quality score between MAF thresholds (ANOVA, $p=0.56$); however, on average there was a small decrease in the quality score (0.6–0.8%) (Additional file 2: Table 9a-c). The parameter values for the number of included reference haplotypes showed statistical differences starting at the contrast ${k}_{hap}=100$ to ${k}_{hap}=1000$, with imputation accuracy decreasing at higher *k*_*hap*_ parameter values (Additional file 2: Table 9d-f).

Improvements in haplogroup concordance was also evaluated using Hi-MC to assign haplogroups. Following imputation, there was a mean increase (31.2–32.5%) in accuracy of haplogroup assignment across different Reference Panel MAF thresholds. However, there was no statistically significant difference between these MAF thresholds (ANOVA, $p=0.83$) (Additional file 2: Table 10a-c). With an increase in the *k*_*hap*_ parameter, a decrease in accurate haplogroup assignment was observed, with contrasts at ${k}_{hap}=100$ to ${k}_{hap}=2500$ becoming statistically significant. These patterns were observed when macrohaplogroups were examined (Additional file 2: Table 11). On average, haplogroup concordance ranged from 16.7 to 21.0%, while macrohaplogroup concordance ranged from 88.0 to 88.4%

Taken together, these findings indicate optimum values of ${k}_{hap}=500$ for the number of reference haplotypes, and MAF > 0.1% for the minor allele frequency threshold of the Reference Panel.

#### Overall microarray performance

Using our recommended settings (${k}_{hap}=500$, MAF > 0.1%), most genotypes were successfully imputed in most cases, with ${\mu }_{MCC}=0.618$ ($95\% confidence interval [CI] = 0.615, 0.620$). The best performing chip was the GSA-24v2-0_A1-b37 ($MCC=0.658;95\%CI=0.636, 0.681$), and the worst performing chip was the HumanOmni2.5S-8v1_B-b37 ($MCC=0.381;95\%CI=\mathrm{0.320,0.441}$) (Additional file 2: Table 12).

On average, macrohaplogroups assigned using HaploGrep2.0 from imputed data were concordant with the truth set 88.2% of the time ($95\%CI=88.1\%,89.4\%$). The GSAMD-24v2-0_20024620_A1-b37 was the best performing microarray dataset in terms of HaploGrep macrohaplogroup concordance ($99.4\%; 95\%CI=99.2\%,99.7\%$), while the InfiniumImmunoArray-24v2-0_A-b37 was the worst performing microarray dataset ($10.8\%;95\%CI=9.6\%,12.0\%$). On average there was an improvement in concordance of 1.5%. HumanOmni2.5S-8v1_B-b37 had the largest improvement (24.4%). HumanOmni5-4v1_B-b37 was the worst performing microarray dataset, with a 13.6% decrease in concordance (Additional file 2: Table 12).

On average, macrohaplogroups assigned using Hi-MC from imputed data were concordant with the truth set 91.8% of the time ($95\%CI=91.7\%,91.9\%$). BDCHP-1X10-HUMANHAP240S_11216501_A-b37 was the best performing microarray dataset in terms of Hi-MC macrohaplogroup concordance ($99.9\%, 95\%CI=99.8\%,100\%)$, and InfiniumOmniZhongHua-8v1-3_A1-b37 was the worst performing ($28.6\%;95\%CI=26.9\%,30.4\%$). The overall increase in improvement was 24.9% (Additional file 2: Table 12), with the HumanOmni5-4v1-1_A-b37 the best performing chip, increasing 43.6%, and HumanOmni1-Quad_v1-0_B-b37 the worst performing, showing a 32.8% decrease in concordance.

#### Overall haplogroup concordance

Concordance of individual haplogroups was estimated at the macro-haplogroup level using HaploGrep2.0 and Hi-MC. Before imputation, less than 50% of sequences from macrohaplogroup V were assigned to their connect macrohaplogroup by HaploGrep2.0 (Additional file 2: Table 13a), and less than 50% of sequences from macrohaplogroups H, HV, I, M, V, W, X were assigned to their correct macrohaplogroup by Hi-MC (Additional file 2: Table 13b). Imputation accuracy as measured by macrohaplogroup concordance using HaploGrep2.0 showed a difference with the microarray datasets ranging from a decrease of 16.6% (HV) to an increase of 52.9% (V). With the exception of L5, all African macrohaplogroups showed a slight decrease (3.12–0.18%). For the Native American-associated macrohaplogroups, only B showed a decrease (5.02%). Among the East Asian-associated macrohaplogroups, G, N, and Z showed a decrease (0.88–7.42%). Among the Euro-Indian-associated macrohaplogroups, H, J, and U showed a decrease (0.14–1.82%). Imputation accuracy as measured by macrohaplogroup concordance using Hi-MC showed a difference with the microarray datasets from a decrease of 15.7% (B) to an increase of 89.9% (M). All African macrohaplogroups showed a slight decrease (8.9–0.64%). The Native American-associated macrohaplogroups, B and C showed a decrease (0.15–15.7%). Among the East Asian-associated macrohaplogroups, only N showed a decrease (6.5%). Among the Euro-Indian-associated macrohaplogroups, only U showed a decrease (0.8%). However, it should be noted that Hi-MC did not detect any presence of macrohaplogroups F, G, L4, L5, Y, or Z.

### Alzheimer’s disease neuroimaging initiative

We applied MitoImpute to data from 258 participants in the ADNI study, who had provided both WGS [5] and microarray data [21] (Additional file 2: Table 14). The ADNI microarray data were mapped to the rCRS and following imputation sites with an IMPUTE2 info score ≤ 0.3 were discarded. Both HaploGrep2 [30] and Hi-MC [31] were used to assign haplogroups to the WGS, microarray, and imputed data. Genotypes were moderately successfully imputed, as measured by MCC (${\mu }_{MCC}=0.322; 95\%CI=\mathrm{0.294,0.350}$). This is in contrast with the microarray dataset for the chip with which ADNI was genotyped (Illumina Human610-Quad BeadChip, Human610-Quadv1_B-b37, ${\mu }_{MCC}=0.606; 95\%CI=\mathrm{0.576,0.637}$).

Using HaploGrep2.0, the correct macrohaplogroup to 95.7% of samples for the microarray data, which improved to 97.7% after imputation. Macrohaplogroup V showed any improvement of 66.7%, whereas all other macrohaplogroups showed no change, with the exception of H, which showed a 0.9% decrease (Additional file 2: Table 15a). The corresponding improvement using Hi-MC was 37.9% to 95.0%. Macrohaplogroups A, H, J, JT, M, N, V, W, and X all showed improvements, ranging from 27.2 to 100% (Additional file 2: Table 15b). The results for macrohaplogroups M, V, W, and X, are particularly noteworthy since they had no correct assignments prior to imputation. Macrohaplogroup HV remained at 0% concordance before and after imputation.

## Discussion

Investigations into the genetic basis of human mitochondrial disease and of evolutionary history rely on the accurate alignment of homologous nucleotide positions, and complete mtDNA sequences [36]. These two factors, in turn, benefit from globally diverse sequences being included in MSAs used in these investigations. The imputation of missing variants can mitigate datasets of incomplete mtSNVs; however, accurate alignment of sequences and consistent placement of gap character states is fraught with difficulty and time consuming for even experienced bioinformaticians [14]. Lack of publicly available reference MSAs and reference panels, therefore, presents a limitation to researchers investigating mitochondrial disease or evolutionary history. We address this limitation by creating a reference MSA from 36,960 globally diverse mtDNA sequences, which was manually curated by experienced researchers to ensure consistency of the placement of gap character states. Aligning novel sequences to the Reference MSA will alleviate the pressures of the alignment process by providing a guide for these new sequences.

The Reference MSA and Reference Panel we present here are globally and phylogenetically representative. Despite less than 20% of samples that we extracted from GenBank having geographic provenance metadata available, the samples that do contain this information suggest there are at least 103 geographic regions from 49 countries that cover all inhabited continents. These include populations usually not represented in major population genetic datasets (e.g., the 1000 Genomes Project), such as Pacific Islanders and Indigenous Australians. Additionally, all PhyloTree [34] macrohaplogroups are present in our Reference MSA and Reference Panel. To the best of our knowledge, this is the largest and most genetically and geographically diverse curated mtDNA reference panel publicly available and readily downloadable.

Additionally, as a curated MSA, the Reference MSA and Reference Panel can be subsampled for use in answering evolutionary and disease-associated research questions. Furthermore, the Reference MSA can be used as a reference panel for the imputation of mtSNVs. This Reference Panel will enable comparison and combined analyses across datasets of differing age and completeness. The Reference Panel has been packaged into a user-friendly mtSNV imputation pipeline, MitoImpute.

We evaluated how accurately we could impute mtSNVs using our Reference Panel, as measured by the concordance of assigned haplogroups and Matthews Correlation Coefficient of genotypes. Across most microarray datasets, we were able to improve genotype concordance and macrohaplogroup assignment marginally when assigned using HaploGrep2.0 and significantly when using Hi-MC. As HaploGrep2.0 already accurately assigns macrohaplogroups, these results suggest we are successfully imputing phylogenetically informative mtSNVs. Some macrohaplogroups experienced marginal decreases in their correct assignment; however, this does not appear to be biased to any locality outside of Africa. As all haplogroups, except for haplogroups JT and X, experienced an average improvement > 30%, this suggests that the Reference Panel is not biased towards improvement for certain lineages over others. The addition of new sequences to the Reference Panel will only further increase accurate haplogroup assignment in populations or mtDNA lineages that are still underrepresented. We also tested the practical use of our Reference Panel by imputing mtSNVs in the ADNI dataset, demonstrating that the Reference Panel and imputation pipeline can successfully impute genotypes and, in some instances, dramatically increase the correct macrohaplogroup assignment. Given that there are 499 samples in the ADNI genotyping dataset that were not re-sequenced in subsequent phases, this demonstrates the utility of our Reference Panel for long-term studies that need to bring their older, incomplete dataset to the same standard as newer, complete datasets.

Performance testing of the MitoImpute pipeline using microarray datasets revealed a seemingly counterintuitive result; the decrease in imputation accuracy as the *k*_*hap*_ parameter increases. Increasing the *k*_*hap*_ parameter increases the number of haplotypes in the reference panel from which IMPUTE2 will impute. We suspect that increasing the number of reference haplotypes beyond 1000 leads to a greater chance of mismatch between the incomplete sample haplotypes and the reference panel haplotypes, particularly in microarray datasets with few mtSNVs. Alternatively, highly diverse reference panels may contain a large number of haplotypes uninformative for imputing variants missing from the study sample, which has previously been noted by [37]. The limitations of the MAF and *k*_*hap*_ parameters, we suspect, is due to a dearth of mtSNVs in some microarray datasets. Datasets with a small number of variants from which to impute missing mtSNVs will always present this limitation, and we recommend users proceed with caution when using these datasets for subsequent analyses.

We did not split the Reference Panel into population-specific or haplogroup-specific sub-panels for numerous reasons. Only 19.3% of GenBank samples had geographic provenance metadata available, which would have significantly limited our ability to utilise the majority of sequences. Additionally, previous studies have noted that large reference panels with diverse haplotypes can improve imputation quality when admixture has occurred [10], such as in post-colonial societies. However, other studies have suggested that ‘global’ reference panels decrease imputation quality, while population-specific reference panels may increase imputation accuracy [38, 39]. With this in mind, users of MitoImpute can tentatively assign haplogroups to their microarray data to use an a priori guide to subsampling sequences from the Reference Panel. However, we note the *k*_*hap*_ parameter should achieve this intuitively and automatically. Further studies could use MitoImpute to investigate whether the full or subsampled Reference Panel achieves greater imputation accuracy on both single-population and multi-population microarray samples.

We aimed to create the Reference MSA with as many as possible completely-sequenced mitochondrial genomes representing as diverse as possible global haplotypes. Our search criteria for harvesting publicly available sequences therefore may exclude some complete sequences with large deletions. Small deletions remain present in the Reference MSA as gap character states; however, insertions were removed to retain the rCRS site numbering convention. We acknowledge these limitations of MitoImpute. Another limitation is that MitoImpute was not designed to detect heteroplasmy, but it can be detected if raw probe intensity data are available [40]. Finally, we acknowledge that resequencing is often the preferable option for dealing with incomplete sequences. However, MitoImpute provides a ready alternative when raw biomaterial is no longer available for resequencing or for the case of limited financial resources.

## Conclusions

Our Reference Panel provides an opportunity for datasets with limited mitochondrial genetic variation to be analysed with a more complete set of genetic variants and a more accurate assignment of haplogroups. The global disparity in medical research is evident in the high proportion of European individuals (~ 78%) association study catalogues [41]. The 1000 Genomes Project phase 3 includes 2504 individuals from 26 populations, however, these individuals were often sampled from 1 to 3 cities within geographically diverse countries, such as China. Our Reference Panel contains sequences from at least 103 regions in at least 49 countries, capturing a more globally-representative sample of mitochondrial genetic diversity. The diversity included in our Reference Panel will allow researchers to perform imputation in under-represented human populations, contributing to solving the disparity in medical genomics research. This study also highlights the imperative to include accurate and detailed metadata when submitting sequences to public repositories, such as GenBank. Having only geographic provenance metadata available for 19.3% of downloaded GenBank sequences limits our ability to determine regions underrepresented in DNA databases. As haplogroups are only useful for determining geographic provenance at a fine sub-haplogroup level [1], haplogroups cannot be relied on as geographic proxies.

## Supplementary Information

**Additional file 1**. Supplementary methods. Includes the search term for downloading sequences from GenBank and the creation of the 2011 reference alignment.

**Additional file 2**. Supplementary tables. Includes sequence IDs, geographic provenance data, haplogroup assignment summaries, and statistical test results.

## Data Availability

The datasets generated and/or analysed during the current study are available at https://github.com/sjfandrews/MitoImpute (https://doi.org/10.5281/zenodo.4338785). Data used in the preparation of this article were obtained from the Alzheimer’s Disease Neuroimaging Initiative (ADNI) database (adni.loni.usc.edu). As such, ADNI investigators contributed to the design and implementation of ADNI and/or provided data but they did not participate in analysis or writing of this report. A list of ADNI investigators can be found at: http://adni.loni.usc.edu/wp-content/uploads/how_to_apply/ADNI_Acknowledgement_List.pdf.

## Abbreviations

ADNI: Alzheimer’s disease neuroimaging initiative
ISMs: In silico microarrays
MAF: Minor allele frequency
MCC: Matthews correlation coefficient
MSA: Multiple sequence alignment
mtDNA: Mitochondrial DNA
mtSNVs: Mitochondrial DNA single nucleotide variants
WGS: Whole-genome sequence
