# Supplementary material for: A globally diverse reference alignment and panel for imputation of mitochondrial DNA variants
Source: BMC Bioinformatics. 2021 Sep 1;22:417. doi: 10.1186/s12859-021-04337-8 (PMC8409003; doi:10.1186/s12859-021-04337-8)
Supplement: Supplementary file 1 — Additional file 1. Supplementary methods. Includes the search term for downloading sequences from GenBank and the creation of the 2011 reference alignment. [file 12859_2021_4337_MOESM1_ESM.docx]

**Supplementary Information****: A globally diverse reference alignment and panel for imputation of mitochondrial DNA variants**

Tim W McInerney^1^, Brian Fulton-Howard^2^, Christopher Patterson^3,4^, Devashi Paliwal^1^, Lars S Jermiin^5,6,7,8^, Hardip R Patel^1^, Judy Pa^3,4^, Russell H Swerdlow^9^, Alison Goate^2^, Simon Easteal^1^, Shea J Andrews^2*^, for the Alzheimer’s Disease Neuroimaging Initiative^†^

^1^John Curtin School of Medical Research, Australian National University, Canberra, Australian Capital Territory, Australia

^2^Ronald M. Loeb Center for Alzheimer’s Disease, Department of Neuroscience, Icahn School of Medicine at Mount Sinai, New York City, NY, USA

^3^Mark and Mary Stevens Neuroimaging and Informatics Institute, Keck School of Medicine, University of Southern California, Los Angeles, CA, USA

^4^Department of Neurology, Alzheimer’s Disease Research Center, Keck School of Medicine, University of Southern California, Los Angeles, CA, USA

^5^CSIRO Land & Water, Commonwealth Scientific Industrial & Research Organization, Acton, ACT 2601, Australia

^6^Research School of Biology, Australian National University, Canberra, ACT 2601, Australia

^7^School of Biology and Environmental Science, University College Dublin, Belfield, Dublin 4, Ireland

^8^Earth Institute, University College Dublin, Belfield, Dublin 4, Ireland

^9^Department of Neurology, Alzheimer’s Disease Center, University of Kansas, Fairway, KS, USA

*Correspondence to: Shea Andrews, The Icahn School of Medicine at Mount Sinai, 1 Gustave L. Levy Place, New York, NY 10029, USA.

Tel: +1-212-659-8632; E-mail: [shea.andrews@mssm.edu](mailto:shea.andrews@mssm.edu)

^†^Data used in the preparation of this article were obtained from the Alzheimer’s Disease Neuroimaging Initiative (ADNI) database (adni.loni.usc.edu). As such, ADNI investigators contributed to the design and implementation of ADNI and/or provided data but they did not participate in analysis or writing of this report. A list of ADNI investigators can be found at:

<http://adni.loni.usc.edu/wp-content/uploads/how_to_apply/ADNI_Acknowledgement_List.pdf>

**Supplementary Methods**

The following search term was used to identify whole human mtDNA sequences from GenBank on 2018-07-18:

(016500[SLEN]:016600[SLEN]) AND Homo[Organism] AND mitochondrion[FILT] AND complete genome NOT (Homo sp. Altai OR Denisova hominin OR neanderthalensis OR heidelbergensis OR consensus OR ancient human remains OR shotgun)

**Reference Alignment**

We used publicly available PhyloTree (van Oven and Kayser, 2009) sequences to create a large (n=7,747) reference alignment with the revised Cambridge Reference Sequence (rCRS) (Andrews et al., 1999) site numbering convention. Inclusion of rCRS in the reference alignment ensures that site numbering conventions are maintained and verified as new sequences are added. We aligned sequences in batches of 50 using the L-INS-i version of MAFFT (Katoh and Standley, 2013), then combined the batches, resolving inconsistent gap placements manually. rCRS site numbers were preserved by removing sites at which gaps were introduced in the rCRS during the alignment process.

**Supplementary tables**

Table S1: Sequences included in the Reference Panel alignment

Table S2: Strand files downloaded from the Wellcome Centre

Table S3: Variable sites found in the Reference Panels at varying minor allele frequencies (MAF) 1%, 0.5%, and 0.1%

Table S4: Summary table of geographic provenance of samples in the reference alignment and panel extracted from GenBank metadata.

Table S5a-f: MCC genotype imputation accuracy across MAF and k_hap_ settings

Table S6a-f: IMPUTE2 info score across MAF and k_hap_ settings

Table S7a-f: HaploGrep2.0 haplogroup concordance across MAF and k_hap_ settings

Table S8a-f: HaploGrep2.0 macrohaplogroup concordance across MAF and k_hap_ settings

Table S9a-f: HaploGrep2.0 haplogroup quality score across MAF and k_hap_ settings

Table S10a-f: Hi-MC haplogroup concordance across MAF and k_hap_ settings

Table S11a-f: Hi-MC macrohaplogroup concordance across MAF and k_hap_ settings

Table S12: Per-chip performance summary using recommended parameter settings (MAF>0.1% and k_hap=500)

Table S13a-b: Proportion of macrohaplogroups correctly assigned using HaploGrep2.0 and Hi-MC before and after imputation

Table S14: ADNI samples with genotype and whole genome sequencing data

Table S15a-b: Macro-haplogroup concordance between genotyped and imputed ADNI data using HaploGrep2.0 and Hi-MC
